# Thermochemotherapy effect of nanosized As_2_O_3_/Fe_3_O_4 _complex on experimental mouse tumors and its influence on the expression of CD44v6, VEGF-C and MMP-9

**DOI:** 10.1186/1472-6750-9-84

**Published:** 2009-10-05

**Authors:** Yiqun Du, Dongsheng Zhang, Hui Liu, Rensheng Lai

**Affiliations:** 1 Department of Pathology and Pathophysiology, School of Basic Medical Sciences, Southeast University, Nanjing, PR China; 2 Department of Pathology, Jiangsu Provincial Hospital of Traditional Chinese Medicine (Nanjing University of Traditional Chinese Medicine Hospital), Nanjing, PR China; 3 Jiangsu Province Key Laboratory of Biomateria and Devices, Southeast University, Nanjing, PR China; 4 Department of laboratory, Nanjing Tongren Hospital,, Nanjing, PR China

## Abstract

**Background:**

Both thermotherapy and arsenic have been shown to be active against a broad spectrum of cancers. To reduce the limitations of conventional thermotherapy, improve therapeutic anticancer activity, reduce the toxicity of arsenic on normal tissue, and increase tissue-specific delivery, we prepared a nanosized As_2_O_3_/Fe_3_O_4 _complex (Fe_3_O_4 _magnetic nanoparticles encapsulated in As_2_O_3_). We assessed the thermodynamic characteristics of this complex and validated the hyperthermia effect, when combined with magnetic fluid hyperthermia (MFH), on xenograft HeLa cells (human cervical cancer cell line) in nude mice. We also measured the effect on the expression of *CD44v6*, *VEGF-C*, and *MMP-9 *which were related to cancer and/or metastasis.

**Results:**

The nanosized As_2_O_3_/Fe_3_O_4 _particles were approximately spherical, had good dispersibility as evidenced by TEM, and an average diameter of about 50 nm. With different concentrations of the nanosized As_2_O_3_/Fe_3_O_4 _complex, the correspondingsuspension of magnetic particles could attain a steady temperature ranging from 42°C to 65°C when placed in AMF for 40 min. Thermochemotherapy with the nanosized As_2_O_3_/Fe_3_O_4 _complex showed a significant inhibitory effect on the mass (88.21%) and volume (91.57%) of xenograft cervical tumors (p < 0.05 for each measurement, compared with control). In addition, thermochemotherapy with the nanosized As_2_O_3_/Fe_3_O_4 _complex significantly inhibited the expression of *CD44v6*, *VEGF-C*, and *MMP-9 *mRNA (p < 0.05 for each).

**Conclusion:**

As_2_O_3_/Fe_3_O_4 _complex combined with MFH had is a promising technique for the minimally invasive elimination of solid tumors and may be have anticancerometastasic effect by inhibiting the expression of *CD44v6*, *VEGF-C*, and *MMP-9*.

## Background

Clinically, heating of certain organs or tissues to temperatures between 41°C-46°C (the procedure is termed tissue "hyperthermia") has been effective in tumor therapy. Presently, traditional thermotherapy protocols utilize radiofrequency waves, microwaves, or lasers, each of which has many limitations. With the development of nanotechnology, magnetic nanoparticles have been used not only as drug carriers but also in tumor hyperthermia, as such particles absorb energy from high frequency AMF. Nanoscaled magnetic fluid has been found to absorb much more energy than conventional materials, and this energy is further transferred to tumor cells resulting in tumor temperatures of 42°C-45°C. This process, termed "Magnetic Fluid Hyperthermia (MFH)" can be used for the treatment of either non-cancer diseases or tumors [1]. Magnetic nanoparticles may be good thermoseeds for localized hyperthermia treatment of cancers [2], permitting the heating of and damage to normal tissue to be avoided, thus overcoming the limitations of conventional heat treatment. Fe_2_O_3 _magnetic nanoparticles used in MFH were reported to have a significant therapeutic effect on xenograft liver cancer in nude mice [3]. Depending on the applied temperature and the duration of heating this treatment either results in direct tumor cell killing or makes the cells more susceptible to concomitant radio- or chemotherapy. Numerous groups are working in this field worldwide, but only one approach has been tested in clinical trials so far.

Metastatic spread of the solid tumor depends on a critical cascade of events, which includes tumor cell adhesion to a distant site, extracellular matrix degradation, migration, proliferation, and ultimately neovascularization. Tumors that produce higher levels of metastasis-related factors, such as proteins encoded by the "cluster of differentiation 44v6" (*CD44v6*) gene, and the genes encoding vascular endothelial growth factor-C (*VEGF-C*) and matrix metalloproteinase-9 (*MMP-9*), may show more aggressive behavior than do tumors negative for these factors. Thus, a treatment that could inhibit the expression of these tumor metastasis-related factors would be of great interest.

Arsenic is a well-documented carcinogen that also appears to be a valuable therapeutic tool in cancer treatment [4]. The first use of As_2_O_3 _in cancer therapy was to treat acute promyelocytic leukemia (APL) [5]. The results of in vitro research and clinical trials have shown that As_2_O_3 _is effective in inhibiting tumor growth, and in inducing the differentiation and apoptosis of APL cells. Because of its significant anti-cancer effects, As_2_O_3 _has been tested in patients with other tumor types, including gastric cancer [6], neuroblastoma [7], esophageal carcinoma [8], and head and neck cancers [9]. Moreover, As_2_O_3 _was shown to inhibit tumor metastasis by reducing the expression of metastasis-related genes [10-12].

The purpose of this study was to prepare nanosized As_2_O_3_/Fe_3_O_4 _complex for tumor thermochemotherapy and validate its effect on xenograft tumor in nude mice as a premature treatment. We also tested the ability of a nanosized As_2_O_3_/Fe_3_O_4 _complex combined with MFH to inhibit the expression of metastasis-associated genes.

## Methods

### Reagents

As_2_O_3 _was purchased from Sigma (St Louis, MO; Lot A1010). A 1 mM stock solution in RMPI 1640 medium (Gibco) was prepared, stored at 0-4°C, and diluted before use. Calf serum from newborn animals was obtained from Si-Ji-Qing Biotechnology Co. (Hangzhou, China). HEPES (the free acid) and trypsin were obtained from Amresco Corp. RNAiso reagent, AMV retroviridase, dNTPs, Oligo(dT)18, *Taq *DNA polymerase, and DNA markers were purchased from Takara Biotechnology Co. (Dalian, China). *VEGF-C*, *CD44v6*, and *MMP-9 *primers were obtained from Shen-neng-bo-cai Biotechnology (Shanghai, China).

### Preparation of the Nanosized As_2_O_3_/Fe_3_O_4 _Complex

Fe_3_O_4 _magnetic nanoparticles were prepared by chemical coprecipitation. Briefly, solutions of FeCl_3_·6H_2_O and FeCl_2_·4H_2_O were mixed, at a molar ratio of iron (II) to iron (III) of about 0.6, under nitrogen purging and with stirring, with ammonia (1.5 mol/L) added dropwise until the pH attained pH 9. A dark precipitate (Fe_3_O_4 _magnetic nanoparticles) appeared rapidly, and stirring was continued for 30 min. After 30 min at 90°C, the Fe_3_O_4 _magnetic nanoparticles were isolated using a permanent magnet and dried in vacuo.

The nanosized As_2_O_3_/Fe_3_O_4 _complex was prepared by an impregnation process. Briefly, Fe_3_O_4 _magnetic nanoparticles were added to a solution of As_2_O_3 _(0.01 mg/mL, pH = 5, adjusted with acetic acid) with sonication. After 30 min of thermal treatment at 80°C, the resulting nanosized As_2_O_3_/Fe_3_O_4 _complex was centrifuged at 2,000 g/min for 10 min, rinsed twice with absolute ethanol, and dried under vacuo. The diameter of the nanosized As_2_O_3_/Fe_3_O_4 _complex particles was measured by transmission electron microscopy (TEM; Hitachi H-600 instrument; Japan).

### Heat Testing of Nanosized As_2_O_3_/Fe_3_O_4 _Complex in Vitro

The nanosized As2O3/Fe3O4 complex was dispersed in 0.9% (w/v) NaCl, at Fe3O4 concentrations of 0.5, 1.0, 1.5, 2.0 and 2.5 mg/mL, and 2 mL aliquots of magnetic fluids were added to flat-bottomed cuvettes and placed in a high frequency electromagnetic field. The distance from the bottom of the cuvette to the center of the source (coils) of the high frequency field was 5 mm. The output frequency was 230 kHz and the output current 20 A. Throughout 1 h of incubation, the temperature was measured every 5 min.

### Cell Culture

HeLa cells (a human cervical cancer cell line, provided by the Institute of Biochemistry and Cell Biology, Shanghai Institute of Biological Sciences, Chinese Academy of Sciences) were maintained in RPMI-1640 medium supplemented with 10% (v/v) heat-inactivated fetal calf serum, 100 units/mL penicillin, and 100 mg/L streptomycin, at 37°C in a 5% CO_2_/95% air (v/v) incubator under 95% relative humidity.

### Animal Experiments

Female BALB/C nude mice, aged 6 weeks, were purchased from the Lakes Animal Experimental Center of the Institute of Biochemistry and Cell Biology, Shanghai Institute of Biological Sciences, China. The animal experiments were approved by the regional animal ethics committee and the mice treated in accordance with the international animal ethics guidelines. All of the mice were maintained in the animal facility of School of Basic Medical Sciences, Southeast University, China. Mice were housed up to 8 animals per cage in individual ventilation cages and fed with specific pathogen-free mice chow *ad libitum*. Exponentially growing HeLa cells (density >10^5 ^cells/mL) were injected subcutaneously around the right posterior limb rump. When tumor diameters reached 0.5 cm, mice were divided into four groups of eight mice each: (1) sterile 0.9% (w/v) NaCl (control group), (2) 5 μM As_2_O_3 _dispersed in 0.9% (w/v) NaCl (As_2_O_3 _group), (3) 1 mg/mL of Fe_3_O_4 _dispersed in 0.9% (w/v) NaCl (Fe_3_O_4 _group), and (4) 1 mg/mL of Fe_3_O_4 _and 5 μM As_2_O_3 _dispersed in 0.9% (w/v) NaCl (As_2_O_3_/Fe_3_O_4 _group). The solution/suspension (1, 2, 3, or 4) were directly injected into tumors with a volume equal to half the tumor volume. In our study, we applied the method of multipoint injection, following clockwise, at the 3, 6, 9, and 12 o'clock points. The tumors of the Fe_3_O_4 _and As_2_O_3_/Fe_3_O_4 _groups were exposed to high frequency AMF (f = 230 kHz, I = 20 A) for 30 min, three times at 24 h intervals [13]. Tumor temperature was measured at multipoint using an infrared thermometer (ZyTemp-TN18 model; China). Mice were sacrificed after 6 weeks, and the mass and volume of each tumor were measured. Tumor growth inhibition was evaluated by measuring mass and volume inhibition proportions. Mass inhibition (IM) was calculated as (1 - Relative tumor mass) × 100%, where relative tumor mass (RTM) was the mean tumor mass of the experimental group divided by the mean tumor mass of the control group. Similarly, volume inhibition (IV) was calculated as (1 - Relative tumor volume) × 100%, where relative tumor volume (RTV) was the mean tumor volume of the experimental group divided by the mean tumor volume of the control group.

### Expression of *CD44V6*, *VEGF-C*, and *MMP-9 *after Thermochemotherapy of HeLa cells

#### In vitro treatment and sampling

HeLa cells were seeded in 50 mL culture flasks at 6 × 10^5 ^cells per flask. After 24 hours, cells were grown in: (1) RPMI1640 medium containing 10% (v/v) fetal calf serum (positive control group); (2) 5 μM or 10 μM As_2_O_3 _(As_2_O_3 _groups); (3) 1 mg/mL of Fe_3_O_4 _magnetic nanoparticles with or without AMF exposure (Fe_3_O_4 _groups); or (4) 1 mg/mL of Fe_3_O_4 _and 5 μM or 10 μM As_2_O_3 _with AMF exposure (As_2_O_3_/Fe_3_O_4 _complex groups), with six flasks used to test each of the above conditions. AMF exposure consisted of electromagnetic exposure for 60 min under high frequency AMF (f = 230 kHz, I = 20 A). All flasks were incubated for 48 hours and cells were thereafter isolated.

#### RNA isolation

RNA was extracted from cells using the RNAiso reagent, according to the supplier's protocol. The purity and concentration of RNA were determined by spectrophotometry at 260 nm. The quality of RNA was checked by electrophoresis of 2-3 μL samples in 1% (w/v) agarose gels, staining with ethidium bromide, and examining the 28S and 18S rRNA bands under UV light. No significant degradation was observed in any RNA sample.

#### Semi-quantitative reverse transcription polymerase chainreaction (RT-PCR)

Total RNA was denaturated for 10 min at 70°C. Each RT reaction (20 μL volume) contained 2 μL of total RNA,1 μL of Oligo dT18, 2 μL of each dNTP (10 mM), 0.5 μL RNasin, 1 μL AMV retroviridase, 4 μL 5 × AMV buffer, and 9.5 μL of DEPC water. Each reaction mixture was incubated for 1 hour at 42°C, and the reverse transcriptase was inactivated by heating at 95°C for 5 min. Each cDNA product was frozen at -20°C until use.

PCR reactions were performed in 25 μL volumes containing 5 μL cDNA, 2 μL 10× PCR buffer, 2 μL dNTP (2.5 mM), 1 μL sense primers, and 1 μL antisense primers [14-16] (20 pmol/μL, please see Table 1; β-actin primers were used as an internal control), 0.5 μL *Taq *DNA polymerase (5 U/μL), 2.5 μL MgCl_2 _(25 mM), and 11 μL DEPC water. After initial denaturation at 96°C for 3 min, the mixtures were subjected to a varying number of denaturation cycles (each at 1 min at 94°C), annealing (1 min at 56°C for β-actin; 57°C for *VEGF-C*; and 60°C for other genes) and extension (2 min at 72°C), and a final extension at 72°C for 10 min, in a PTC-100 thermal cycler (MJ Research, Watertown, MA). Samples were stored at -20°C. For each reaction, a negative control employed distilled water instead of cDNA, and cDNA from untreated HeLa cells served as the positive control.

**Table 1:** PCR primers and PCR conditions.

**Gene**	**Primers**	**Tm (°C)**	**No. of cycles**	**Product size (bp)**	**Reference**
*CD44v6*	5'-GACACATATTGCTTCAATGCTTCAGC-3'	60	35	348	[14]
	5'-TACTAGGAGTTGCCTGGATGGTAG-3'				
*VEGF-C*	5'-AGACTCAATGCATGCCACG-3'	57	35	435	[15]
	5'-TTGAGTCATCTCCAGCATCC-3'				

*MMP-9*	5'-GTGCTGGGCTGCTGCTTTGCTG-3'	60	35	303	[15]
	5'-GTCGCCCTCAAAGGTTTGGAAT-3'				
*β-actin*	5'-CGTCTGGACCTGGCTGGCCGGGACC-3'	56	28	600	[16]
	5'-CATGAAGCATTTGCGGTGGACGATG-3'				

Amplified PCR products were electrophoresed with DNA markers, on 2% (w/v) agarose gels containing ethidium bromide. Bands were visualized under UV light and each gel image was captured by a digital camera. Imagetool 2.0 software was used for semi-quantitative analysis of electrophoresis results.

## Results

### Characteristics of the Nanosized As_2_O_3_/Fe_3_O_4 _Complex

**Figure 1:**
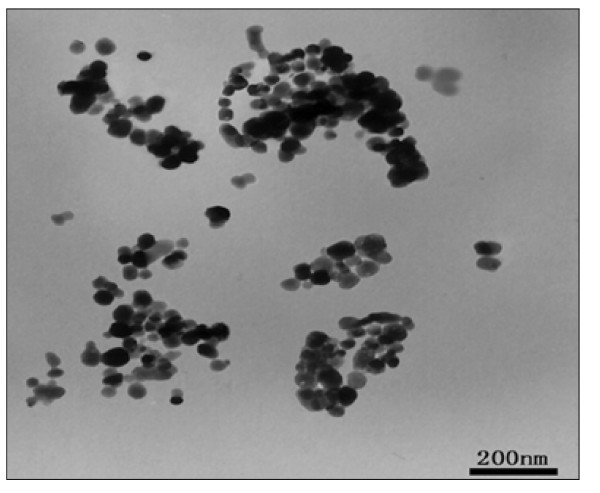
**TEM image of the nanosized As_2_O_3_/Fe_3_O_4 _complexes**.

**Figure 2:**
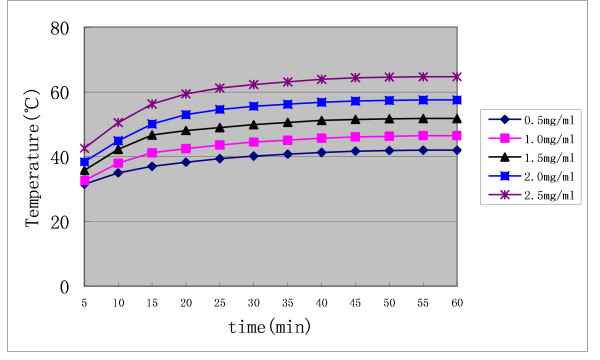
**Heating test curve of nanosized As_2_O_3_/Fe_3_O_4 _complex fluid in AMF in vitro**. Output frequency = 230 kHz, output current = 20A; the concentrations of Fe3O4 were 0.5, 1.0, 1.5, 2.0, and 2.5 mg/mL.

The nanosized As_2_O_3_/Fe_3_O_4 _complex particles were approximately spherical, uniform in size, and had good dispersibility, with an average diameter by TEM of about 50 nm (Figure 1) [17]. Upon dispersion in 0.9% (w/v) NaCl and exposure to high frequency AMF (output frequency 230 kHz, output current 20 A) for 60 min, the nanosized As_2_O_3_/Fe_3_O_4 _complexes increased the temperature of magnetic fluid (MF) from 42°C to 65°C, depending on the concentration of Fe_3_O_4 _(Figure 2), with a permanent change seen after 40 min of electromagnetic exposure. As a 1 mg/mL concentration of Fe_3_O_4 _in the As_2_O_3_/Fe_3_O_4 _complex increased the MF temperature to 47°C, we chose this Fe_3_O_4 _concentration for further experiments.

### In Vivo Inhibitory Effects of the As_2_O_3_/Fe_3_O_4 _Complex and AMF exposure on Xenograft Cervical Cancer in Nude Mice

**Figure 3:**
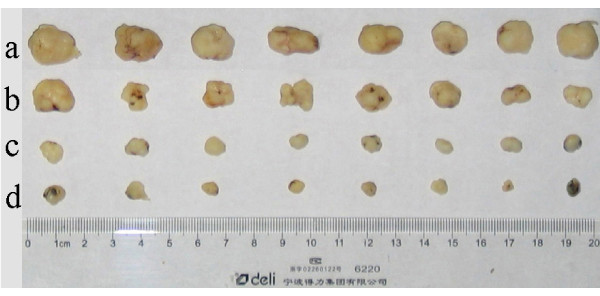
**Morphological changes of xenograft tumors in nude mice treated by different methods. a**. control group (sterile 0.9% [w/v] NaCl); **b**. As_2_O_3 _group (5 μM As_2_O_3 _dispersed in 0.9% [w/v] NaCl); **c**. Fe_3_O_4 _group (1 mg/mL of Fe_3_O_4 _magnetic nanoparticles dispersed in 0.9% [w/v] NaCl plus MFH); **d**. As_2_O_3_/Fe_3_O_4 _group (1 mg/mL of Fe_3_O_4 _and 5 μM As_2_O_3 _dispersed in 0.9% [w/v] NaCl plus MFH). The tumors become smaller in animals treated with protocols '**a**' to '**d**'. Compared with the control, each group showed significant reductions in tumor volume and mass (p < 0.05 for both).

**Figure 4:**
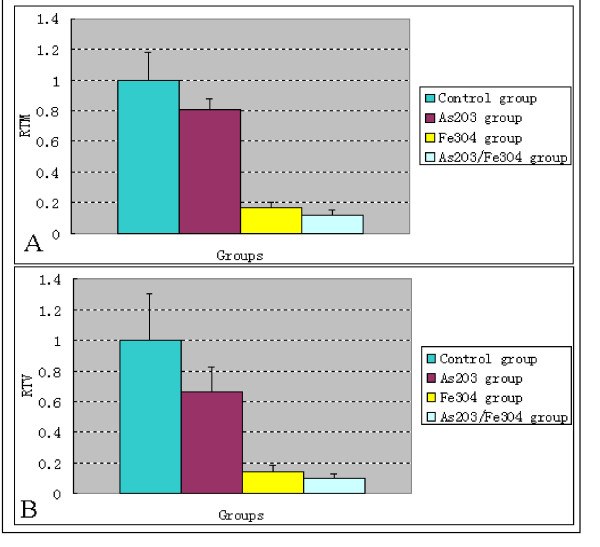
**Part (A): Relative tumor mass (RTM) of xenograft cervical cancer in nude mice after treatment. Part (B): Relative tumor volume (RTV) of xenograft cervical cancer in nude mice after treatment**. These measurements were obtained 6 weeks after the start of treatment.

**Figure 5:**
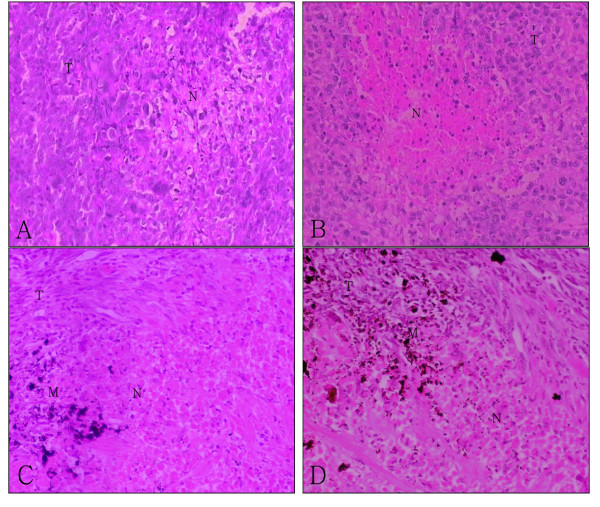
**Histological changes in xenograft tumors (H & E) (×200)**. Sections from the (A) control, (B) As_2_O_3_, (C) Fe_3_O_4_, and (D) As_2_O_3_/Fe_3_O_4 _groups are shown (T, tumor tissue; N, necrosis; M, materials of Fe_3_O_4 _or As_2_O_3_/Fe_3_O_4_. Histological examination revealed that the necrotic areas in **B**, **C**, and **D **were much larger than the intrinsic necrosis of the tumor shown in **A**.

Following intratumor injection of simple Fe_3_O_4 _magnetic nanoparticles or nanosized As_2_O_3_/Fe_3_O_4 _complexes, and exposure to high frequency AMF (f = 230 kHz, I = 20 A) for 30 min, almost the entire tumor got heated by the nanoparticles and the temperature of tumors rose to 44°C-45°C. Compared with control tumors, the tumors of all experimental groups were smaller (Figure 3 &4). The mass and volume inhibition rates in the As_2_O_3_/Fe_3_O_4 _group were 88.21% and 91.57%, respectively, significantly higher than observed in the control, the As_2_O_3_, and the Fe_3_O_4 _groups (p < 0.05 for each, Table 2). Histological examination revealed that, in both the As_2_O_3_/Fe_3_O_4 _and Fe_3_O_4 _groups, many black nanoparticles accumulated in the stroma of the tumors, with widespread tumour necrosis surrounding the nanoparticles. The necrotic areas of the As_2_O_3 _group were larger than those of the control group (Figure 5).

Presently, clinical thermotherapy induces substantial damage to surrounding healthy tissues. We applied a multipoint injection strategy, injecting nanoparticles into the 3, 6, 9, and 12 o'clock points of each tumor, to ensure almost whole tumor to get homogeneously heated and minimize damage to surrounding tissue. Thus, because of the targeted and localized thermogenic activity of MFH, normal tissue without magnetic particles should not be damaged. We found that, because of the dual activity (chemotherapy and hyperthermia) of the nanoparticles, the therapeutic effect of Fe_3_O_4 _magnetic nano-microspheres containing As_2_O_3_, in combination with AMF exposure, was greater than that of Fe_3_O_4 _magnetic nanoparticles combined with AMF exposure, and that of As_2_O_3 _treatment alone.

**Table 2:** Extent of volume and mass inhibition of xenograft cervical cancer in nude mice after treatment.

**Group**	**Tumor volume (mm^3^)** **( ± S, n = 8)**	**Tumor mass (g)** **( ± S, n = 8)**	**Volume inhibition (%)**	**Mass inhibition (%)**
Control group	931.51 ± 284.26	0.789 ± 0.143	0	0
Single As_2_O_3 _group	616.94 ± 147.96^(1)^	0.634 ± 0.056^(1)^	33.77	19.65
Single Fe_3_O_4 _magnetic nanoparticles group	132.79 ± 36.30^(1)^	0.128 ± 0.029^(1)^	85.74	83.78
Nanosized As_2_O_3_/Fe_3_O_4 _complex group	78.50 ± 32.73^(1)^	0.093 ± 0.028^(1)^	91.57	88.21

(1) p < 0.05 vs. control group. These measurements were obtained 6 weeks after the start of treatment.

### Semi-quantitative RT-PCR

**Figure 6:**
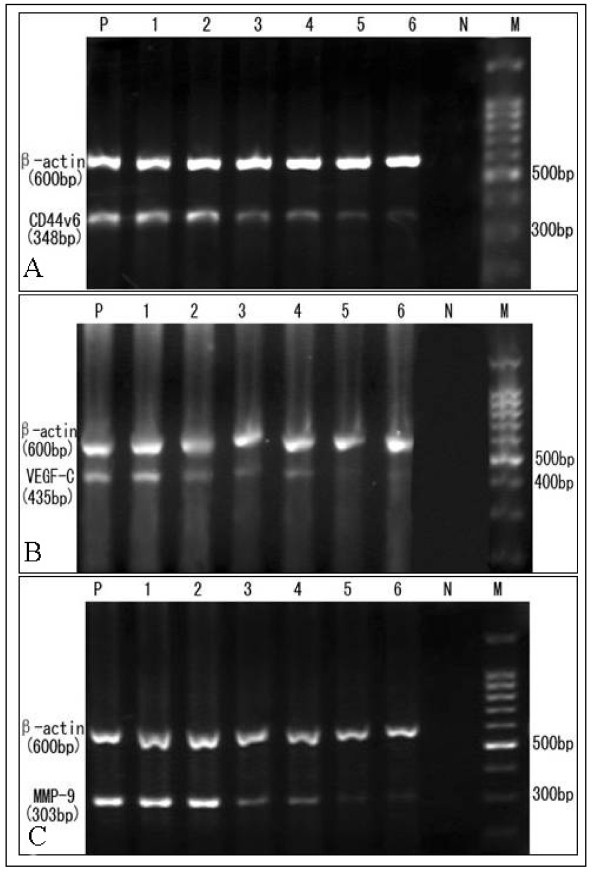
**Expression of (A) *CD44v6*, (B) *VEGF-C*, and (C) *MMP-9 *mRNA after treatment of HeLa cells**. Lanes: **P**, positive control (untreated HeLa cells); **1**, Fe_3_O_4 _without MFH; **2**, Fe_3_O_4 _plus MFH; **3**, nanosized As_2_O_3 _(5 μM)/Fe_3_O_4 _plus MFH; **4**, 5 μM As_2_O_3_; **5**, nanosized As_2_O_3 _(10 μM)/Fe_3_O_4 _plus MFH; **6**, 10 μM As_2_O_3_;**N**, negative control; **M**, DNA marker.

HeLa cells exposed to free As_2_O_3 _or to nanosized As_2_O_3_/Fe_3_O_4 _complexes under AMF showed dose-dependent inhibition of *CD44v6*, *VEGF-C*, and *MMP-9 *mRNA synthesis, which indicated that As_2_O_3 _can inhibit *CD44v6*, *VEGF-C *and *MMP-9 *mRNA expression. In contrast, exposure to Fe_3_O_4 _magnetic nanoparticles and AMF inhibited expression of *VEGF-C*, but not of *CD44v6 *and *MMP-9 *(Figure 6, Table 3).

**Table 3:** Results of semi-quantitative RT-PCR analysis

	**relative gray value (gene/*β*-actin), ( ± S)**						
	
**Gene**	**P**	**1**	**2**	**3**	**4**	**5**	**6**
*CD44v6*	0.386 ± 0.049	0.396 ± 0.052	0.438 ± 0.115	0.210 ± 0.075^(1)^	0.244 ± 0.120^(1)^	0.142 ± 0.064^(1)(2)^	0.117 ± 0.030^(1)(3)^
*VEGF-C*	0.605 ± 0.166	0.514 ± 0.083	0.398 ± 0.079^(1)^	0.305 ± 0.132^(1)(2)^	0.344 ± 0.038^(1)^	0.095 ± 0.016^(1)(3)^	0.150 ± 0.036^(1)^
*MMP-9*	0.966 ± 0.205	1.053 ± 0.222	0.997 ± 0.093	0.461 ± 0.170^(1)^	0.466 ± 0.121^(1)^	0.244 ± 0.107^(1)(2)^	0.220 ± 0.098^(1)(3)^

*CD44v6: *(1) p < 0.05 *vs*. **P**, (2) p < 0.05 *vs*. **3**, (3) p < 0.05 *vs*. **4**; *VEGF-C: *(1) p < 0.05 *vs*. **P**, (2) p < 0.05 *vs*. **2 **and **4**, (3) p < 0.05 *vs*. **6**; *MMP-9: *(1) p < 0.05 *vs*. **P**, (2) p < 0.05 *vs*. **3**, (3) p < 0.05 *vs*. **4**.

**P**, positive control (untreated HeLa cells); **1**, Fe_3_O_4 _without MFH; **2**, Fe_3_O_4 _plus MFH; **3**, nanosized As_2_O_3 _(5 μM)/Fe_3_O_4 _plus MFH; **4**, 5 μM As_2_O_3_; **5**, nanosized As_2_O_3 _(10 μM)/Fe_3_O_4 _plus MFH group; **6**, 10 μM As_2_O_3_.

## Discussion

Heat therapies such as hyperthermia and thermoablation are very promising approaches in the treatment of cancer. Compared with available hyperthermia modalities, MFH yields better results in uniform heating of the deeply situated tumors. In this approach, fluid consisting of superparamagnetic nanoparticles (magnetic fluid) is delivered to the tumor. An AMF is then used to heat the particles and the corresponding tumor, thereby ablating it[18]. Interstitial hyperthermia following direct injection of nanoparticles has also been proven successful in many animal models. Jordan A et al. reported good effects of MFH on C3H mammary carcinoma in vivo[19]. A variety of magnetic fluids have been applied using related techniques (differing mostly in the formulation of the nanoparticles), but so far only few of these approaches has been successfully translated from research to clinical stage.

All magnetic nanoparticles used so far in vivo are composed of the iron oxides magnetite (Fe_3_O_4_) and maghemite (γ-Fe_2_O_3_) due to their low toxicity and their known pathways of metabolism. As demonstrated by X-ray diffraction, Fe_3_O_4 _nanoparticles are of the spinel type and magnetic Fe_3_O_4 _[17,20] can absorb a considerable amount of energy and transform this energy into heat when exposed to AMF. We successfully prepared nanosized As_2_O_3_/Fe_3_O_4 _complexes using co-precipitation and impregnation processes. These particles were successful in the warming of tumors upon exposure to high frequency AMF, suggesting their usefulness in hyperthermia protocols. By adjusting the concentration of Fe_3_O_4_, we could select a suitable temperature (42°C-46°C) for tumor hyperthermia. At a 1 mg/mL Fe_3_O_4 _concentration in the As_2_O_3_/Fe_3_O_4 _complex, the temperature of the magnetic fluid rose to 47°C after exposure to high frequency AMF for 30 min.

Nanosized As_2_O_3_/Fe_3_O_4 _complexes, combined with MFH, had a significant therapeutic effect on xenograft tumor in nude mice, by inducing tumor cell apoptosis and inhibiting cell growth. We injected complexes directly into the tumor tissue, rather than into healthy tissue at the tumor boundary, thus delivering nanoparticles to the desired regions. Such nanoparticles may be internalized into tumor cells and organelles [19], and transmission electron microscopy showed that the nanosized As_2_O_3_/Fe_3_O_4 _complex particles were indeed so internalized, both into cells and into organelles such as the nucleoli and lysosomes [13]. Although the mechanism by which nanoparticles enter tumor cells is not yet known, pinocytosis or penetration may be in play, as the nanoparticle diameter is small. Moreover, hyperthermia may increase the permeability of biological membranes. These findings suggest that the As_2_O_3_/Fe_3_O_4 _composite nanoparticles may be useful as thermoseeds for localized hyperthermia treatment of cancers, without damaging normal tissue, thus overcoming the limitations of conventional heat treatment. Moreover, by using these complexes, As_2_O_3 _can be localized to tumor tissue, thus reducing the As_2_O_3 _toxicity to normal tissue. This method may therefore allow a combination of localized thermogenic and chemotherapeutic activity, even in tumors located deep inside the body, simultaneously minimizing damage caused by heat and toxicity to normal tissue surrounding the tumor. The MFH also can act as a temperature control switch in vivo by controlling the concentration of Fe_3_O_4_, thus minimizing the risk of overheating during therapy. In comparison with traditional hyperthermia, the double function (chemotherapy and hyperthermia) and targeted activity of the combination of nanosized As_2_O_3_/Fe_3_O_4 _complexes and MFH are most important characteristics. Based on the data in Table 2 and Figure 4, although the tumor inhibition appears to be primarily due to the heat, we can still see that the combination was more effective than either Fe_3_O_4 _magnetic nanoparticles plus MFH, or As_2_O_3 _treatment alone. In addition, the heat created during thermotherapy may directly damage tumor cells or may make cells more susceptible to accompanying chemotherapy [21].

MFH has been undergoing clinical testing. In three clinical studies the safety and the feasibility of the technique has been proven. In March 2003, the first clinical feasibility study on magnetic nanoparticle hyperthermia was started with 14 patients suffering from glioblastoma multiforme [22]. All patients received a neuro-navigationally guided injection of the magnetic fluid into the tumor. The amount of fluid and the spatial distribution of the depots were planned in advance by means of a specially developed treatment planning software following magnetic resonance imaging (MRI). The actually achieved magnetic fluid distribution was measured by computed tomography (CT).

In prostate cancer, a direct injection technique of MFH was investigated in phase-I-study. The feasibility and good tolerability was shown in the trial[23,24]. This novel approach requires specific tools for planning, quality control and thermal monitoring, based on appropriate imaging and modelling techniques. Treatment planning was carried out using CT of the prostate. Nanoparticle suspensions were injected transperineally into the prostate under transrectal ultrasound and flouroscopy guidance.

Another prospective feasibility study enrolled 22 patients with proven recurrences and residual tumors (non-resectable and pre-treated, e.g. prostate and cervix carcinoma, soft tissue sarcoma)[25]. Three implantation methods were established: Infiltration under CT fluoroscopy (group A), TRUS (transrectal ultrasound)--guided implantation with X-fluoroscopy (group B) and intra-operative infiltration under visual control (group C). In group A and B the distribution of the nanoparticles can be planned prior to implantation on the basis of three-dimensional image datasets. These approaches of MFH above were well tolerated by patients.

MFH is suitable for treatment of superficial tumors by direct injection of nanoparticle suspension, which was first reported in 1997 [19]. For deep tumors, the feasibility and good tolerability of different injection techniques (CT, ultrasound or X-fluoroscopy guided) were shown. Endoscope such as Oesophagoscope and vaginoscope can be respectively used as guiding devices for esophageal cancer and cervical cancer. A different approach of using magnetic nanoparticles for heating tumor cells is termed ferromagnetic embolization hyperthermia. This technique, which uses a feeding artery to carry nanoparticles into the tumor, seems to be especially well suited for the treatment of hepatic malignancies due to the differences in blood supply between hepatic tumor cells and normal liver parenchyma. Several preclinical studies on arterial embolization hyperthermia of liver cancer were reported [26-28], and Granov reported this treatment on clinical use[29].

Metastasis, a unique characteristic of malignant tumors, is the most difficult issue in the clinical treatment of cancer and the major cause of death. Hyperthermia may trigger immune responses that inhibit the metastasis of graft tumors [30].

"Cluster of differentiation 44" (*CD44*) is a cell surface adhesion molecule that recognizes hyaluronate and mediates diverse functions, such as cell-cell and cell-matrix adhesion, lymphocyte homing, and T-cell adhesion and activation. *CD44 *exists as a standard form (*CD44s*) and as multiple isoforms, each generated by alternative splicing of up to 10 variant exons encoding parts of the extracellular domain. *CD44*, especially the *CD44v6 *variant, has a role in tumor progression and metastasis in human cancers [31]. Expression of *CD44v6 *has been found to correlate significantly with lymphatic and/or hematogenous metastasis. Following the administration of As_2_O_3 _to SGC9701 cells, expression of *CD44 *decreased, suggesting that As_2_O_3 _inhibited *CD44 *expression [32].

In the vascular endothelial growth factor (*VEGF*) family, *VEGF-C *is a ligand for *VEGF *receptor *(VEGFR)-3*, a tyrosine kinase receptor that is predominantly expressed in the endothelium of lymphatic vessels. *VEGF-C *plays an important role in tumor metastasis by mediating the formation of lymphatic vessels. Most cancer cells express *VEGF-C*, and such expression positively correlates with tumor angiogenesis and metastasis [33]. As_2_O_3 _has been reported to inhibit *VEGF *expression, preventing the proliferation, invasion, and metastasis of solid tumors [34]. In addition, expression of *VEGF *mRNA and protein by fibrosarcomas was significantly inhibited after thermotherapy at 42°C [35]. Moreover, the density of tumor vessels in gliomas was reduced after thermotherapy and even more after thermochemotherapy, indicating that thermotherapy may inhibit the expression of *VEGF *mRNA and protein [36].

Matrix metalloproteinases (*MMPs*) are a family of zinc- and calcium-dependent enzymes that degrade extracellular matrix proteins, such as laminin, fibronectin, and collagen. *MMPs *mediate the invasive properties of most malignant cells and promote angiogenesis through their ability to degrade basement membranes and to remodel extracellular matrix architecture. In particular, *MMP-9 *specifically degrades collagen IV, the major component of the basement membrane. Expression of *MMP-9 *has been found to correlate with tumor invasiveness and metastasis, including lymph node metastasis[37]. As_2_O_3 _reduced the expression of *MMP-9 *by nasopharyngeal carcinoma cells in vitro, and decreased their invasive and metastatic properties [12]. Using As_2_O_3_/Fe_3_O_4 _nanoparticles, we found that As_2_O_3 _dose-dependently inhibited the expression of *CD44v6*, *VEGF-C*, and *MMP-9 *mRNA. Moreover, thermotherapy alone inhibited the expression of *VEGF-C *mRNA to some extent.

## Conclusion

The nanosized As_2_O_3_/Fe_3_O_4 _complexes described here possess not only the chemotherapeutic activity of As_2_O_3 _but also the characteristic of strong magnetic responsiveness arising from the presence of Fe_3_O_4_. These particles are suitable for treating superficial tumors by direct injection and for deep tumors by using different injection techniques (CT, ultrasound or X-fluoroscopy guided, intraoperatively under visual control), because they absorb energy from high frequency AMF and also have chemotherapeutic effects. For early stage tumors, thermochemotherapy of nanosized As_2_O_3_/Fe_3_O_4 _complexes could be used as preoperative treatment because of reducing tumor volume and intraoperative bleeding, creating better conditions for latter surgery. In addition, it may be can inhibit tumor metastasis to some extent.

Most researches of MFH in this field applied so far have only been evaluated in preclinical studies. In spite of some Phase I trials have been carried out, it is still too early to claim therapeutic advantages for the applied method because survival benefit or time to progression were not defined endpoints of the finished feasibility studies. However, the ongoing Phase II trials will provide an initial indication whether MFH can improve survival and/or quality of life.

## Abbreviations

MFH: magnetic fluid hyperthermia; AMF: alternating magnetic field; APL: acute promyelocytic leukemia; TEM: transmission electron microscope; HeLa cells: human cervical cancer cell line; RTV: Relative Tumor Volume; RTM: Relative Tumor Mass; RT-PCR: Reverse transcription polymerase chain reaction; *CD44*: Cluster of differentiation 44; *VEGF*: vascular endothelial growth factor; *MMPs*: Matrix metalloproteinases; MRI: magnetic resonance imaging; CT: computed tomography; TRUS: transrectal ultrasound.

## Authors' contributions

YD was responsible for experimental design and completion of all laboratory work presented in this article. DZ contributed to the conception of the study and participated in all stages of the work. HL and RL helped to plan and coordinate the study and helped draft the manuscript. All authors have read and approved the final manuscript.
